# Prevalence of injected drug use and access to OAT in prison: survey in 7 EU countries, 2014-2018

**DOI:** 10.1093/eurpub/ckac131.416

**Published:** 2022-10-25

**Authors:** S Mazzilli, L Royuela, L Tavoschi, L Vandam, L Montanari

**Affiliations:** 1 Scuola Normale Superiore, Pisa, Italy; 2 Department of Translational Research in Medicine, University of Pisa, Pisa, Italy; 3 Public Health Unit, European Monitoring Centre for Drugs and Drug Addiction, Lisbon, Portugal

## Abstract

**Background:**

People in prison report high rates of drug use and drug-related problems and people who use or inject drugs (PWUD) have higher rates of offending and an increased likelihood of spending part of their lives in prison. They represent a vulnerable population with high burden of diseases, socio-economic disadvantages and limited access to healthcare. In this study, we aimed to describe the lifetime prevalence (LTP) of heroin use, injected drug use and opioid agonist therapy (OAT) among people living in prison.

**Methods:**

Individual data collection was carried out in seven European countries (Czech Republic, Latvia, Lithuania, Poland, Portugal, Slovenia, Spain) between 2014 and 2018 with a model European Questionnaire on Drug use among people in Prison. Risk factors analysis was carried out using multivariate logistic regression model.

**Results:**

The analysis of EQDP data found that the LTP of heroin use was overall 22.4% (ranged from 7.3% in Poland to 27.5% in Spain). Female, recidivist offenders, individuals aged below 44 and with an history of mental health condition had a higher likelihood of being heroin user (p-value<0.05). The LTP of injected drug use was overall 20.6%(2176/10,587), while the prevalence of injected drug use in prison was 8.1%(745/9273) (with higher prevalence in countries that did not allow OAT initiation in prison: 17.5% in Latvia, 22.1% in Lithuania and 26.7% in Czech Republic). Among those who used heroin, 44.5%(772/1735) had access to OAT and among them 95.8(566/591) had access to OAT in prison.

**Conclusions:**

Despite the heterogeneity of the results, there is a clear evidence that people in prison have a high prevalence of heroin and injected drug use. Prisons are an important point of access to OAT, and where treatment is available there is a reduction in risk behaviour. It is also important to implement tailored preventive interventions among vulnerable social groups at the community level.

**Key messages:**

• People in prison in Europe have a high prevalence of heroin and injected drug use. Prisons can represent a point of access to integrated prison-community healthcare and social services for PWUD.

• In order to plan adequate healthcare interventions, there is an urgent need to assess and monitor the prevalence of drug use also in other European countries.

